# Intravital Microscopic Evaluation of the Effects of a CXCR2 Antagonist in a Model of Liver Ischemia Reperfusion Injury in Mice

**DOI:** 10.3389/fimmu.2017.01917

**Published:** 2018-01-05

**Authors:** Thiago Henrique Caldeira de Oliveira, Pedro Elias Marques, Fariba Poosti, Pieter Ruytinx, Flávio Almeida Amaral, Laura Brandolini, Marcello Allegretti, Paul Proost, Mauro Martins Teixeira

**Affiliations:** ^1^Immunopharmacology Laboratory, Department of Biochemistry and Immunology, Institute of Biological Sciences, Federal University of Minas Gerais, Belo Horizonte, Brazil; ^2^Laboratory of Molecular Immunology, Department of Microbiology and Immunology, Rega Institute, Catholic University of Leuven, Leuven, Belgium; ^3^Program in Cell Biology, The Hospital of Sick Children, Toronto, ON, Canada; ^4^Dompé farmaceutici S.p.A., Milano, Italy

**Keywords:** hepatic ischemia–reperfusion, chemokines, CXCR2, neutrophil-mediated liver injury, neutrophil migration

## Abstract

**Background:**

Ischemia–reperfusion (IR) is a major contributor to graft rejection after liver transplantation. During IR injury, an intense inflammatory process occurs in the liver. Neutrophils are considered central players in the events that lead to liver injury. CXC chemokines mediate hepatic inflammation following reperfusion. However, few studies have demonstrated in real-time the behavior of recruited neutrophils. We used confocal intravital microscopy (IVM) to image neutrophil migration in the liver and to analyze in real-time parameters of neutrophil recruitment in the inflamed tissue in animals treated or not with reparixin, an allosteric antagonist of CXCR1/2 receptors.

**Materials and methods:**

WT and LysM-eGFP mice treated with reparixin or saline were subjected to 60 min of ischemia followed by different times of reperfusion. Mice received Sytox orange intravenously to show necrotic DNA in IVM. The effect of reparixin on parameters of local and systemic reperfusion-induced injury was also investigated.

**Results:**

IR induced liver injury and inflammation, as evidenced by high levels of alanine aminotransferase and myeloperoxidase activity, chemokine and cytokine production, and histological outcome. Treatment with reparixin significantly decreased neutrophil influx. Moreover, reparixin effectively suppressed the increase in serum concentrations of TNF-α, IL-6, and CCL3, and the reperfusion-associated tissue damage. The number of neutrophils in the liver increased between 6 and 24 h of reperfusion, whereas the distance traveled, velocity, neutrophil size and shape, and cluster formation reached a maximum 6 h after reperfusion and then decreased gradually. *In vivo* imaging revealed that reparixin significantly decreased neutrophil infiltration and movement and displacement of recruited cells. Moreover, neutrophils had a smaller size and less elongated shape in treated mice.

**Conclusion:**

Imaging of the liver by confocal IVM was successfully implemented to describe neutrophil behavior *in vivo* during liver injury by IR. Treatment with reparixin decreased not only the recruitment of neutrophils in tissues but also their activation state and capacity to migrate within the liver. CXCR1/2 antagonists may be a promising therapy for patients undergoing liver transplantation.

## Introduction

Liver ischemia–reperfusion (IR) injury is a complication of several systemic pathologies, including trauma and shock. During liver transplantation, there is always some degree of damage associated with hepatic IR (1–3). Indeed, IR remains a major problem in clinical transplantation, representing more than 10% of early transplant failures and leading to higher incidence of both acute and chronic rejection (4–6). In the ischemic liver, an imbalance of metabolic supply and demand results in severe tissue hypoxia and microvascular dysfunction (7–9). However, liver damage and failure are caused mainly by the reperfusion period, during which there is a shift from metabolic distress caused by ischemia to an excessive immune response triggered by reperfusion (10).

Neutrophils are recruited and activated during reperfusion in several organs, including the liver. These cells are considered central players in the events that lead to injury following reperfusion (11). Once the neutrophils reach the site of inflammation, they release oxidants and proteases that directly injure hepatocytes and endothelial cells, and promote obstruction of hepatic sinusoids, resulting in hepatic hypoperfusion (12). The chemotaxis of these activated neutrophils to the liver parenchyma is directed by a gradient of CXC chemokines. After tissue damage, chemokines are locally secreted by parenchymal cells and resident leukocytes, thereby creating a gradient along which neutrophils can migrate from blood vessels to the site of inflammation. In mice and rats, the chemokines keratinocyte chemoattractant (KC or CXCL1), macrophage inflammatory protein-2 (MIP-2 or CXCL2), and granulocyte chemotactic protein-2 (CXCL6) are important chemoattractants for neutrophils (13–19). These chemokines are recognized by the receptors CXCR1/2 expressed on the neutrophil surface and mediate neutrophil recruitment (20–23). Several authors have shown that the expression of chemokines is required to guide neutrophil migration toward the injury (3, 24, 25). Thus, the blockade of chemokines as well as their receptors might be a valid strategy for the treatment of liver injury induced by the IR. In this context, reparixin (DF1681B) is a chemical derivative of phenyl propionic acids that acts as allosteric antagonist of the chemokine receptors CXCR1 and CXCR2 (26).

Given that leukocytes must enter the site of inflammation from the vasculature, leukocyte recruitment has been a major focus of investigation over many years. Few studies have demonstrated how neutrophils behave within the tissue during injury development (10, 25, 27). The aim of this study was to investigate neutrophil behavior in the presence and the absence of a CXCR1/2 antagonist in animals subjected to liver IR injury. To this end, we used intravital microscopy (IVM) to analyze in real-time parameters of movement, displacement, speed, and directionality of neutrophils in the inflamed liver with or without the CXCR1/2 inhibitor reparixin.

## Materials and Methods

### Mice

Male C57BL/6J and Lysm-eGFP mice (8–12 weeks old) were obtained from the Central Animal Facility of the Universidade Federal de Minas Gerais (UFMG, Brazil). In some experiments, male C57BL/6J mice (8–12 weeks old) were provided by the Animal Facility of KU Leuven (Belgium). The animals were maintained with filtered water and food *ad libitum* in a 12-h dark–light cycle in the thermoneutral zone for mice. All experiments were approved by the animal ethics committee of UFMG (CETEA/UFMG 422/15) and the ethical committee for animal experiments from KU Leuven (P111/2016).

### Hepatic IR Injury Model

The IR was performed as described (28). Mice were anesthetized with an intraperitoneal injection of xylazine (4 mg/kg) and ketamine (80 mg/kg). After a midline laparotomy, mice underwent a sham control operation or IR. In the IR group, the pedicle of the left and median lobes of the liver, containing the bile duct, hepatic artery, and portal vein (comprising 70% of the liver) was occluded using an atraumatic clamp (Aleamed, Kontich, Belgium). After 60 min of ischemia, the clamp was removed and reperfusion was initiated. The following time points were examined after reperfusion: 1, 3, 6, 12, 24, and 48 h. The control operation was performed using the same protocol but without vascular occlusion. In this case, the sham group refers to animals operated in the earliest time-point evaluated in each experiment (1–6 h), since we observed that there was no difference between sham groups at any time-point after surgery, in any of the parameters evaluated (data not shown). Mice were placed on a heating pad to maintain body temperature at 37°C throughout the procedure. Blood was collected for analysis of serum alanine aminotransferase (ALT) as an index of hepatocellular injury using a kinetic test (Bioclin, Belo Horizonte, Brazil). Cytokines and chemokines were quantified by enzyme-linked immunosorbent assay kits (R&D Systems, Minneapolis, MN, USA) both in serum and tissues and real-time quantitative polymerase chain reaction (qPCR) of livers. Fragments of liver were fixed and sectioned for histology as described below. Indocyanine green (ICG; Sigma) clearance by the liver was estimated in serum after injecting a single dose of 20 mg/kg intravenously. Blood was collected 20 min after injection and the amount of ICG was determined by spectrophotometry (absorbance in 800 nm).

### Neutrophil Accumulation in Liver and Lungs

Neutrophil accumulation was determined by liver myeloperoxidase (MPO) content. Fifty milligrams of tissue were homogenized in a buffered solution containing antiproteases, as previously described (29). MPO levels were assessed using 25 µl of the supernatant of the homogenized sample and 25 µl of a solution of 1.6 mM of 3,3′-5,5′-tetramethylbenzidine (Sigma—dissolved in dimethyl sulfoxide) and 0.01 mM of H_2_O_2_, dissolved in phosphate buffer (pH 5.4) containing hexa trimethylammonium bromide (29).

### Measurement of Gene Expression by Real-time qPCR

Relative changes in gene expression were evaluated by qPCRs. Total RNA extraction was performed with the RNeasy Mini Kit (Qiagen, Venlo, The Netherlands) according to the manufacturer’s protocol. Afterward, RNA quantification was achieved using the Nanodrop2000 (Thermo Scientific, Waltham, MA, USA) and for each sample 2 µg of total RNA was reverse transcribed into cDNA using the High-Capacity cDNA Reverse Transcription Kit (Applied Biosystems, Foster City, CA, USA). Relative changes in gene expression were evaluated by qPCR using the TaqMan Fast Universal PCR master mix (Applied Biosystems). Sample mixes were loaded on a 96-well MicroAmp plates (Applied Biosystems) and were analyzed on the 7500 Fast Real-time PCR system. Obtained *Ct* values were processed following the 2^−ΔΔ^*^Ct^* method, with GAPDH serving as housekeeping gene (30).

### Histological Analysis

The livers were washed with 0.9% NaCl and fixed in 4% buffered formalin. Subsequently, the samples were dehydrated in ethyl alcohol solutions, bathed in xylol, and included in histological paraffin blocks. Tissue sections of 5 µm thickness were obtained using a microtome and stained with hematoxylin and eosin. The slices were visualized using the BX41 (Olympus) optical microscope and images obtained using the Moticam 2500 camera (Motic) and Motic Image Plus 2.0ML software.

### Imaging of the Liver Using Confocal Intravital Microscopy (IVM)

Parameters of neutrophil accumulation in the liver were observed using IVM as previously described, using a Nikon Ti C2 confocal microscope equipped with a 10× objective in male mice expressing the green fluorescent protein eGFP mainly in neutrophils (Lysm-eGFP) (31). To optimize imaging of neutrophils, the microscope was set for imaging of GFP-bright cells, excluding low-GFP expressing mononuclear cells (32). After different reperfusion times, the mice were anesthetized and the liver exposed on acrylic support compatible with the microscope. Before image acquisition, the mice were injected i.v. with 100 µl of the fluorophore Sytox Orange (1 µM, Invitrogen) to stain DNA. Neutrophils were counted and their tracking parameters set using the Volocity program (PerkinElmer), which allows to identify and count neutrophils in the videos, frame by frame. This provides quantitative information about neutrophil location, migration distance, velocity, total displacement, and meandering. This information was plotted as mean ± SE of the events in the video, and one video was made per mouse. Each video took 30 min and was recorded at the rate of 1 frame/min.

### Immunofluorescence Microscopy Analysis

Study of histological changes was performed on 4-µm acetone-fixed frozen sections. To investigate neutrophil infiltration in inflamed liver, immunofluorescent labeling was performed using PE rat anti-Mouse Ly6G (Cat: 551461, BD Bioscience, San Jose, CA, USA). Hoechst was used for nuclear counterstaining, and sections were coverslipped with Prolong Gold antifade reagent (Ref: P36934, Life Technologies, Eugene, OR, USA). Images were taken with a Zeiss Axiovert 200M (Carl Zeiss AG, Oberkochen, Germany) and AxioVision Rel 4.8 acquisition software.

### Effect of Reparixin on IR-Induced Liver Injury

The animals were treated with reparixin (DF1681B) provided by Dompé Pharmaceutical S.p.A. The drug was diluted in sterile 0.9% NaCl solution at a dose of 15 mg/kg. The treatment started 15 min before the reperfusion *via* intravenous injection and was continued subcutaneously every 2 until 12 h after the reperfusion. This schedule of treatment has been chosen because of the short half-life of reparixin (33).

### Statistical Analysis

Experimental data analysis was performed with one-way analysis of variance (with Tukey’s *post hoc* test) and Student’s *t*-test provided by Prism 6.0 software (GraphPad). All data are given as the mean ± SEM. *In vivo* experimental groups had at least four mice per group. Data shown are representative of at least two independent experiments. Differences were considered significant at *p* < 0.05.

## Results

### IR Induces Liver Injury and Inflammation

**Figure 1:**
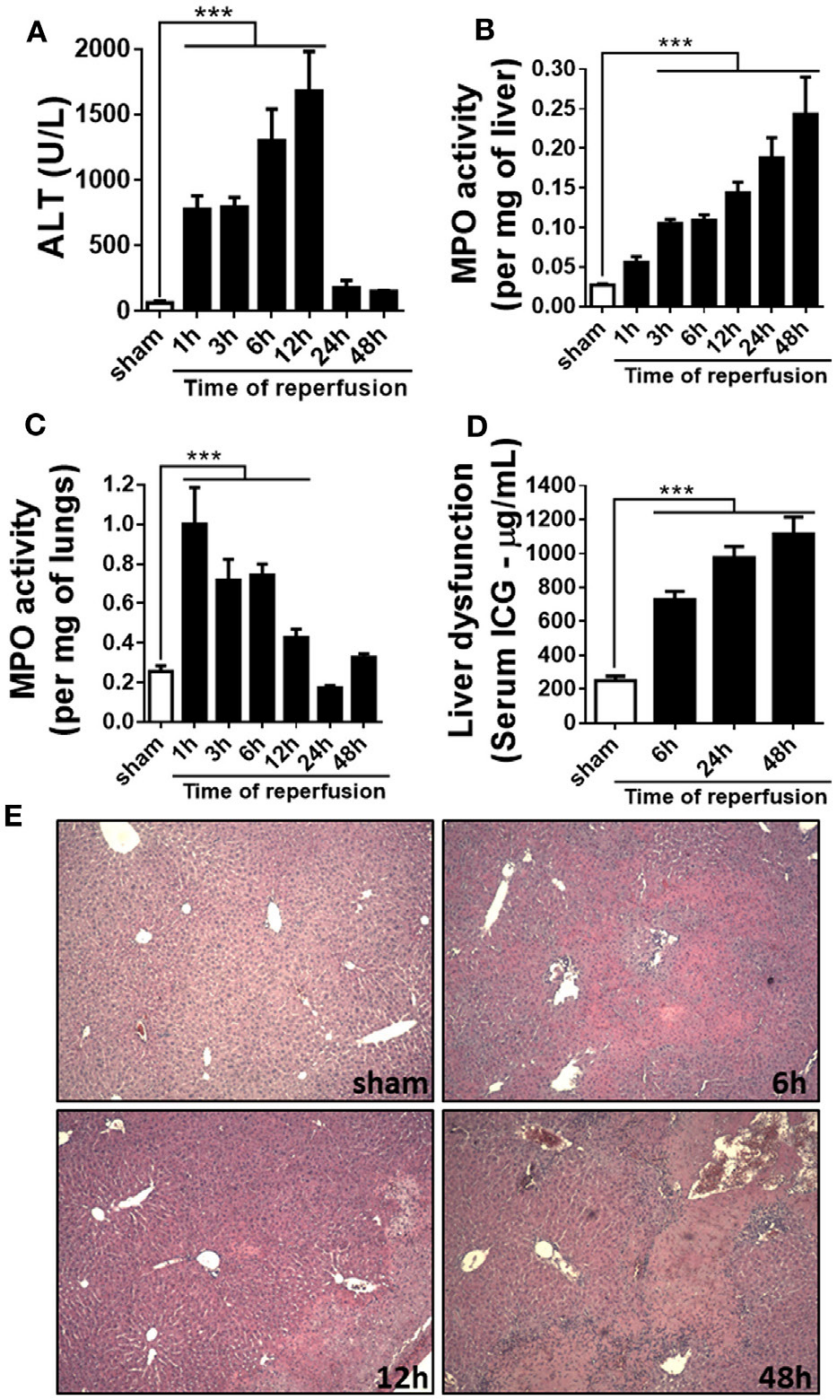
Parameters of liver injury and inflammation were evaluated at different times after reperfusion. The ischemia–reperfusion (IR) induced a significant increase in alanine aminotransferase (ALT) in serum **(A)** and myeloperoxidase (MPO) activity in the liver and lungs **(B,C)** and a significant liver dysfunction in a time-dependent way, increasing up to 48 h after the start of the reperfusion **(D)**. The histological sections showed a significant increased lesion in the liver of animals subjected to IR, mainly at 48 h of reperfusion when compared with sham-operated animals **(E)**. ***p < 0.05 vs with sham-operated animals.

In order to investigate the kinetics of liver inflammation and neutrophil infiltration, mice were subjected to 1 h of ischemia and different times of reperfusion, in which parameters of liver injury and inflammation were evaluated. Hepatic IR caused significant liver damage, as shown by the increase of serum ALT level that reached a peak 12 h after reperfusion (Figure 1A). Furthermore, MPO, one of the main components of neutrophil primary granules, was significantly increased in the liver (Figure 1B). MPO activity increased early and reached a maximum 48 h after reperfusion. Liver IR also triggered remote lung inflammation, as shown by increased MPO levels in the lungs (Figure 1C). To better define the clinical relevance of our procedures, we evaluated the liver metabolic function through the hepatic clearance of ICG, which is a test generally used to evaluate liver function in the clinic. Delay in clearance of ICG is correlated with loss of liver function. As observed in Figure 1D, IR led to an evident liver dysfunction in a time-dependent way, increasing up to 48 h of reperfusion in comparison with sham animals. Moreover, increased ALT level and MPO activity were associated with significant parenchymal cell damage, as observed by elevated sinusoidal congestion and extensive areas of necrosis, mainly present 48 h after reperfusion (Figure 1E). Conversely, control livers showed normal architecture and perfusion, as assessed by tissue histology.

### Liver Injury Caused Intense Chemokine and Cytokine Production

**Figure 2:**
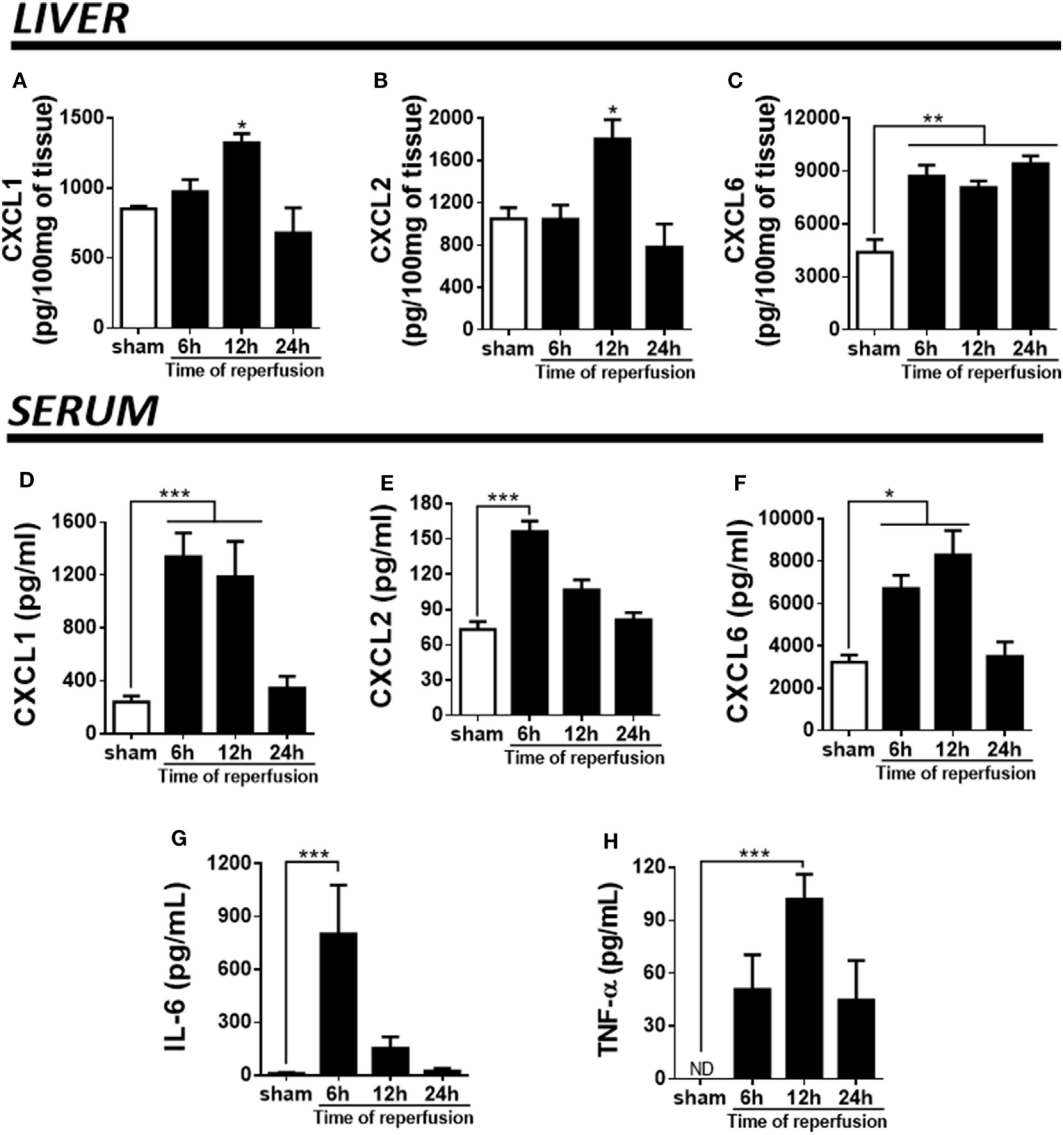
Ischemia–reperfusion induced a significant production of pro-inflammatory markers (detected by enzyme-linked immunosorbent assay) during different times of reperfusion. The liver injury was associated with a high production of CXCL1 **(A)**, CXCL2 **(B)**, and CXCL6 **(C)** in the liver tissue. Similarly, a significant production of CXCL1 **(D)**, CXCL2 **(E)**, CXCL6 **(F)**, IL-6 **(G)**, and TNF-α **(H)** was detected in serum, mainly between 6 and 12 h of reperfusion when compared with sham-operated animals. *p < 0.05, **p < 0.01, and ***p < 0.001 vs with sham-operated animals.

Pro-inflammatory cytokines and chemokines have been implicated in liver IR injury (34). Here, we wished to determine which CXC chemokines with neutrophil attractant properties were being produced in the liver, as well as how IR altered the systemic levels of these mediators. We quantified chemokines in serum and liver extracts along different times of reperfusion. IR induced higher levels of CXCL1, CXCL2, and CXCL6 in mice. Tissue levels of CXCL1 and CXCL2 were maximal 12 h after reperfusion (Figures 2A,B), while CXCL6 was found elevated since the earliest time-point (Figure 2C). In serum, the production of CXCL1, CXCL2, and CXCL6 reached the highest level between 6 and 12 h of reperfusion, returning to near baseline levels after 24 h (Figures 2D–F). Moreover, IL-6 and TNF-α, typical pro-inflammatory cytokines, were significantly up-regulated in serum of mice subjected to IR at 6 and 12 h after reperfusion, respectively (Figures 2G,H).

### Neutrophil Influx during Liver Injury Development Observed by Confocal Intravital Microscopy

**Figure 3:**
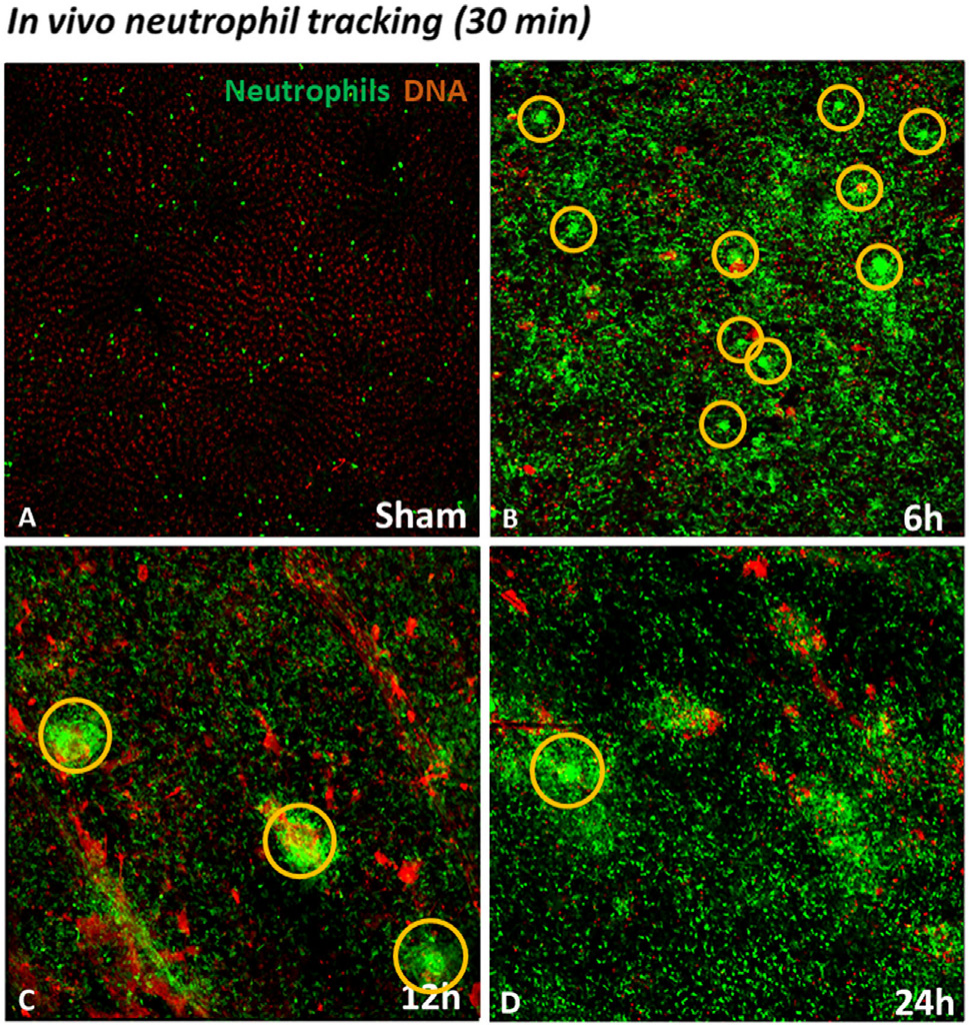
Neutrophil tracking during liver injury development observed by confocal IVM. Sytox orange was injected i.v. to observe the presence of DNA as an indicator of cell death. Low-magnification IVM showed few neutrophils and no presence of DNA in the field **(A)**. Ischemia–reperfusion (IR) induced a huge infiltration of neutrophils in the liver between 6 and 24 h of reperfusion (Lysm-eGFP-expressing cells). In addition, IR caused cell death, observed by hepatic DNA accumulation (orange), in comparison with sham-operated mice **(B–D)**. Scale bars: 100 µm.

In order to establish a general view of neutrophil recruitment to the injured liver, we used mice expressing GFP in neutrophils (lysm-eGFP). To simultaneously evaluate areas of dead cells, we injected mice with the extracellular DNA dye Sytox orange. As shown in Figure 3, in the sham group only a few neutrophils are visible. Furthermore, there was no tissue damage as demonstrated by the absence of extracellular DNA labeling (Figure 3A; see Video S1 in Supplementary Material). On the contrary, a large neutrophil infiltrate was observed in the IR groups 6, 12, and 24 h after reperfusion. In addition, neutrophils could be seen forming clusters in the liver at different times of reperfusion. The presence of dead cells (in orange) was observed in all time points, indicating IR-induced hepatic cell death (Figures 3B–D; see Videos S2–S4 in Supplementary Material).

### Neutrophil Activation and Polarization in Inflamed Tissue

**Figure 4:**
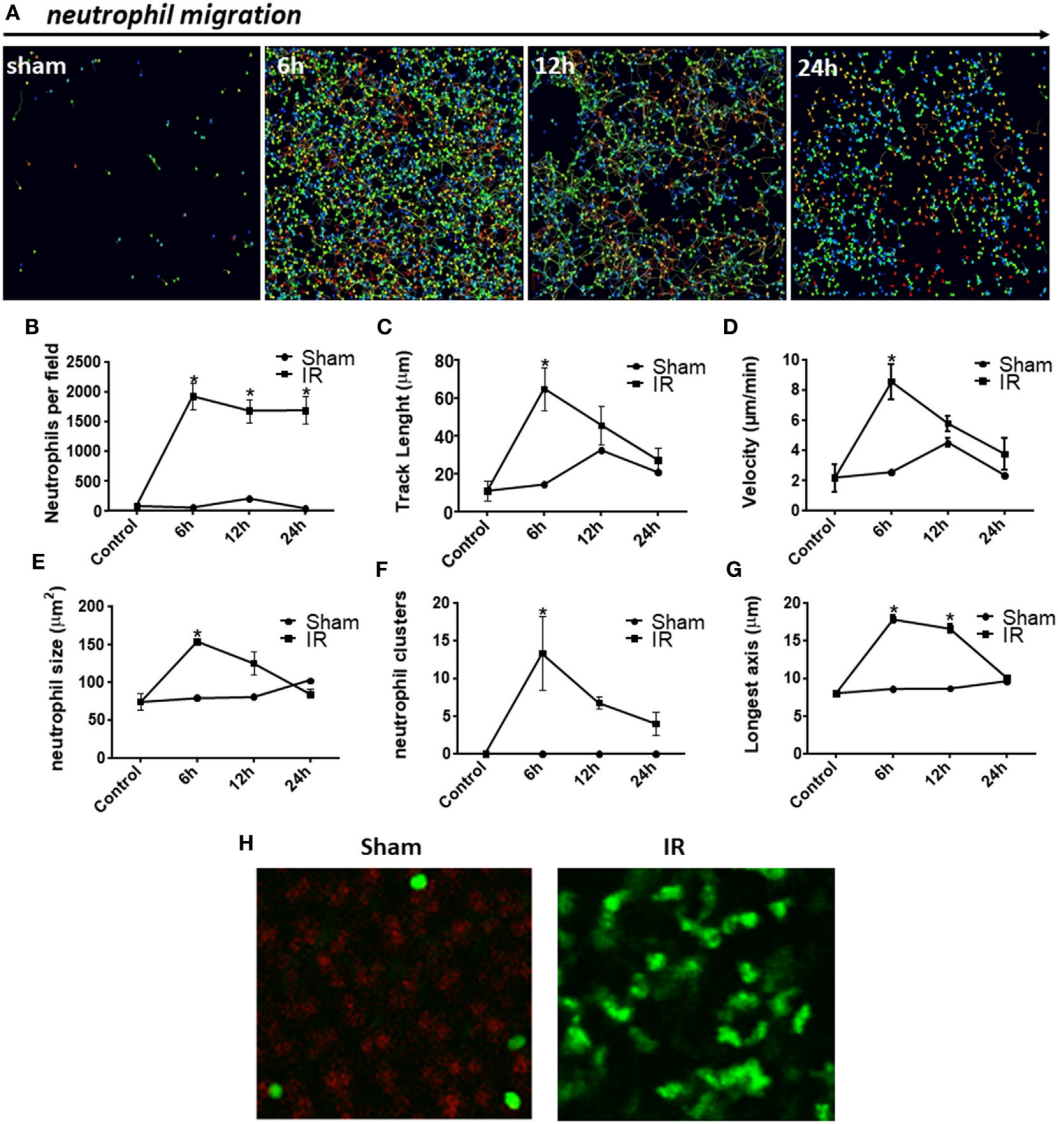
Neutrophil behavior during injury development observed by confocal intravital microscopy. There was neutrophil accumulation in the liver parenchyma of ischemia–reperfusion (IR) mice when compared with sham controls **(A)**. Digital cell tracking showed that IR induced a huge infiltration of neutrophils into the liver **(B)**. Furthermore, after 6 h of reperfusion, the total distance traveled by neutrophils was significantly higher **(C)** and these cells moved faster **(D)**. Indeed IR promoted neutrophil activation as observed by their larger size **(E)**, number of clusters of neutrophils **(F)**, and finally, alteration in shape resulting in more elongated neutrophils **(G,H)**, when compared with sham-operated animals (tracking of neutrophils during 30 min). *p < 0.05 vs sham-operated animals.

We next studied neutrophil movement in the liver parenchyma to evaluate how neutrophils behaved during development of IR injury. As previously noted, there was neutrophil accumulation in the liver parenchyma of IR mice when compared with sham controls. We observed that neutrophils presented significant differences in the pattern and intensity of migration during the reperfusion phase, as illustrated in Figure 4A. Individual neutrophil tracks in the liver tissue are indicated as rainbow-colored traces, which increase in density and length during the reperfusion phase. Interestingly, there was no difference in the number of neutrophils per field among 6, 12, and 24 h of reperfusion when using IVM counting (Figure 4B). However, hepatic neutrophils traveled longer distances and used a higher crawling velocity in the liver 6 h after reperfusion (Figures 4C,D). IR promoted neutrophil activation as observed by their larger size, neutrophil cluster formation, and polarization, which yielded more elongated neutrophils (Figures 4E–H). This activation phenotype, occurring mainly at 6 h of reperfusion, preceded severe liver inflammation and necrosis. Lastly, all neutrophil movement parameters were decreased after 12 h of reperfusion, indicating that the process of neutrophil activation and recruitment started to resolve.

### Reparixin Significantly Reduces Liver IR Injury

**Figure 5:**
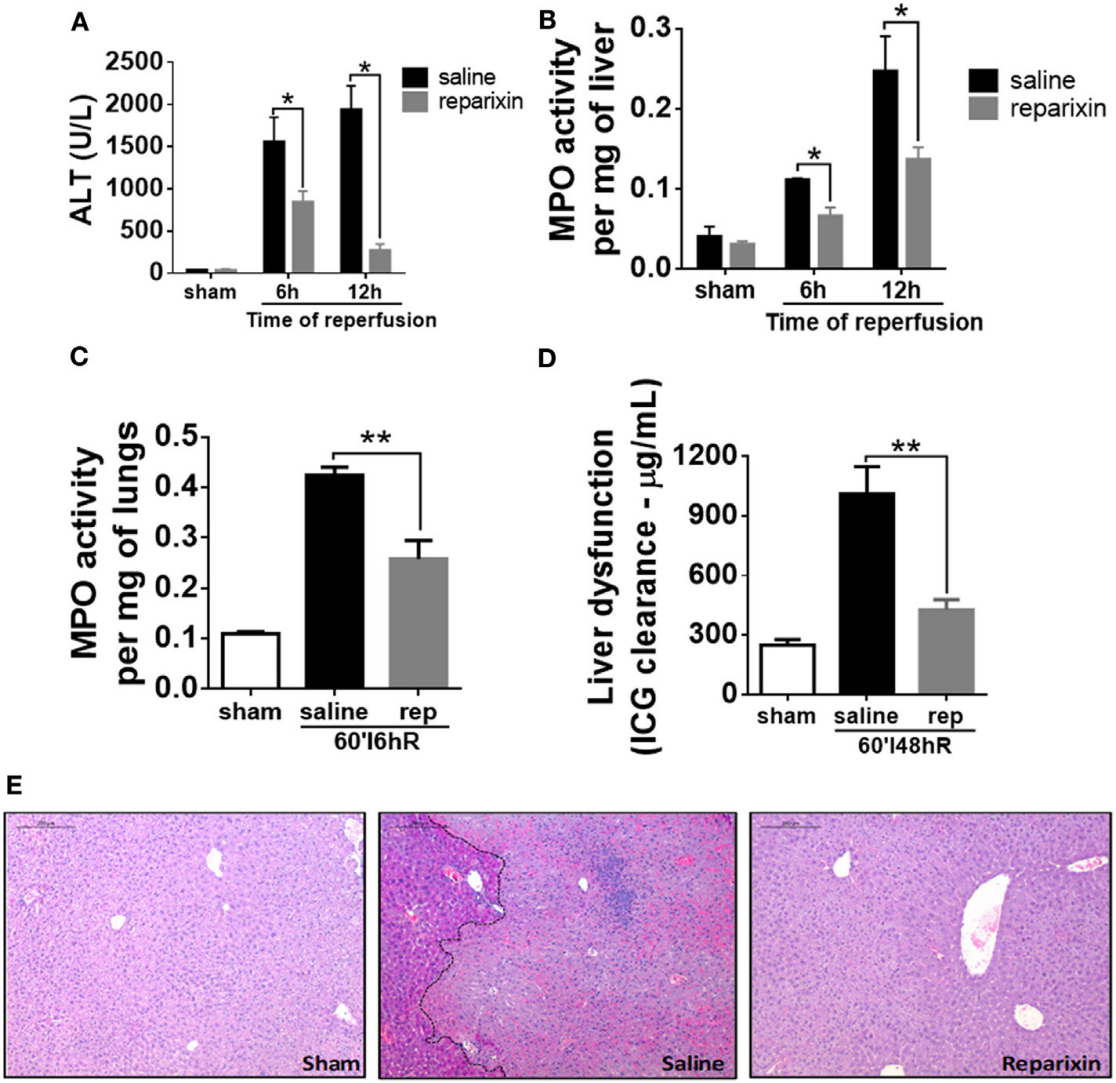
Effects of the treatment with reparixin on levels of alanine aminotransferase (ALT) and myeloperoxidase (MPO) in the liver and lungs of mice subjected to ischemia–reperfusion (IR). Mice received a dose (i.v.) of reparixin (15 mg/kg) 15 min before the reperfusion and every 2 h later (s.c.). Reparixin treatment showed reduced ALT release **(A)**, MPO activity in the liver **(B)** and lungs **(C)**, and faster clearance of indocyanine green **(D)** when compared with animals subjected to IR and treated with saline. *p < 0.05 and **p < 0.01 vs saline-treated livers. **(E)** Representative hematoxylin and eosin staining of livers 12-h post-IR injury. Reparixin-treated animals showed significant histological preservation, contrasting with saline-treated animals, which showed elevated signs of necrosis and intense infiltration of leukocytes.

The main chemokine receptors on neutrophils, which regulate neutrophil recruitment to the site of injury, are CXCR1 and CXCR2. To evaluate the role of CXCR1/2 in liver IR injury, mice were treated with an allosteric antagonist for these receptors, reparixin (DF1681B, 15 mg/kg/i.v.), 15 min before the reperfusion and subsequently every 2 h. As noted, pharmacological inhibition of CXCR1/2 by reparixin significantly reduced liver damage, as observed by reduced ALT in serum (Figure 5A). Moreover, mice treated with reparixin showed reduced neutrophil recruitment to the liver and lungs by approximately 50% as shown by MPO activity (Figures 5B,C). We also observed better liver function in reparixin-treated mice, as assessed by the reduced levels of ICG in the serum (Figure 5D). Histological analysis of the liver of mice subjected to liver IR showed significant sinusoidal congestion, extensive areas of necrosis and infiltration of inflammatory cells (Figure 5E). In reparixin-treated mice, liver architecture was greatly preserved, which coincides with the decrease in ALT and MPO activities (Figure 5E).

### Blocking of CXCR2 Impaired Neutrophil Accumulation during Liver IR Injury

**Figure 6:**
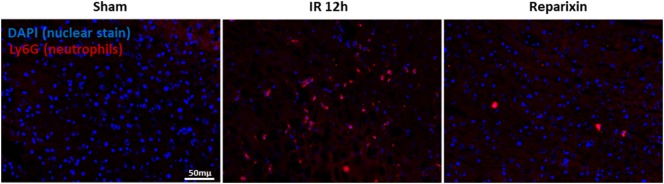
Effects of the reparixin treatment on neutrophil infiltration in liver ischemia–reperfusion (IR) injury. Representative photomicrographs of Ly6G staining (neutrophil marker) and nuclei staining (DAPI) in sham (left), wild-type mice subjected to IR (middle), or IR-injured mice treated with reparixin (right). Accumulation of neutrophil was increased in IR liver compared with sham livers. Mice receiving reparixin showed a significant reduction in the number of infiltrated neutrophils. Ly6G is shown in red and nuclei in blue (×200).

Ly6G is expressed primarily in neutrophils and correlates with the cellular level of differentiation and maturation (35). We next aimed to determine whether CXCR1/2 antagonism could directly affect neutrophil numbers in the IR injured liver. Liver sections of sham-operated mice were negative for Ly6G (Figure 6, left). However, Ly6G positive cells were predominantly detected 12 h after reperfusion in saline-treated mice (Figure 6, middle). Of note, livers of mice treated with reparixin showed significantly less Ly6G staining at 12 h after reperfusion (Figure 6, right).

### Inflammatory Mediator Production Was Significantly Inhibited in Reparixin-Treated Mice

**Figure 7:**
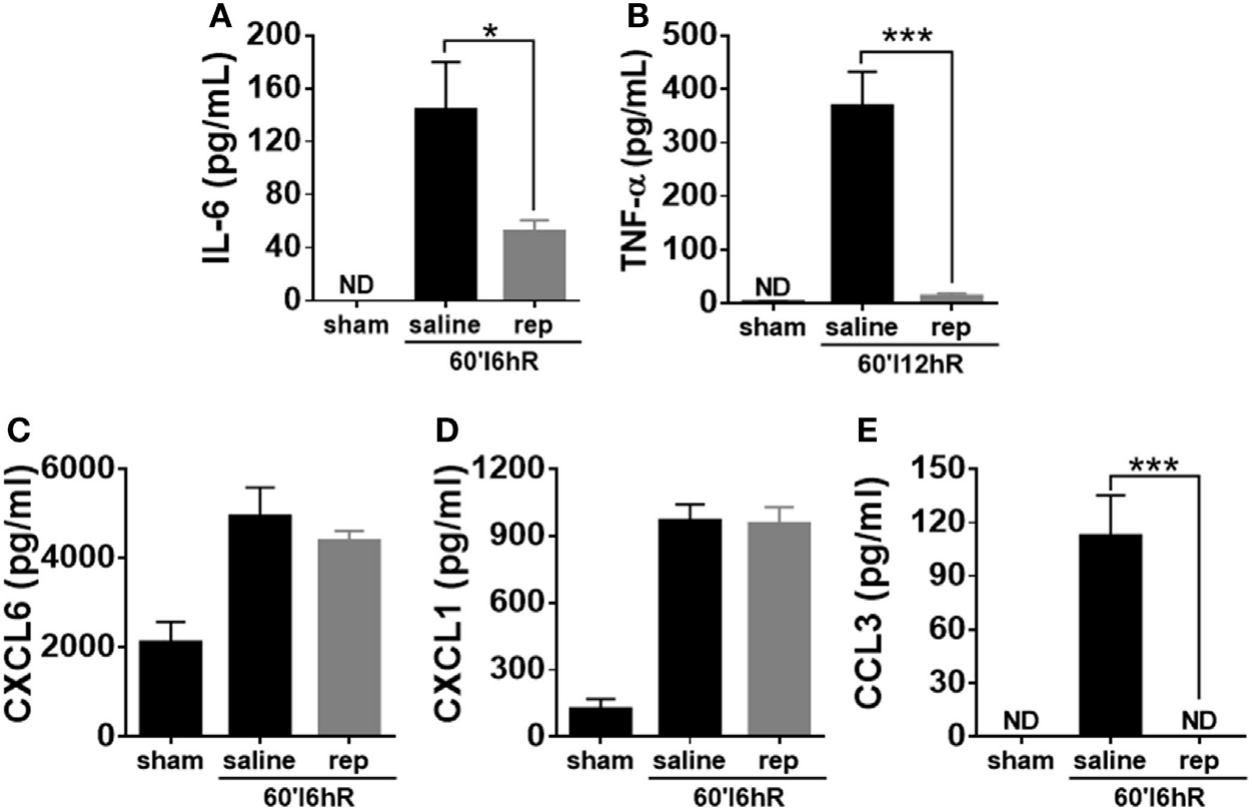
Effects of the treatment with reparixin on the concentrations of cytokines and chemokines in the serum of mice subjected to ischemia–reperfusion (IR). Mice received a dose of reparixin (15 mg/kg) 15 min before the reperfusion (i.v.) and every 2 h later (s.c.). Reparixin reduced the production of the cytokines TNF-α **(A)** and IL-6 **(B)** when compared with animals subjected to IR and treated with saline. No differences in the concentration of CXCL1 **(C)** and CXCL6 **(D)** between mice treated with saline and reparixin was detected. However, mice treated with reparixin showed a lower production of the chemokine CCL3 **(E)** *p < 0.05 and ***p < 0.001 vs relative to saline-treated livers.

To determine whether the inhibition of CXCR1/2 could alter systemic levels of cytokines and chemokines, we quantified the concentration of these mediators in serum of control and reparixin-treated mice. The serum levels of TNF-α were significantly increased at 12 h of IR, while IL-6 and CCL3 were significantly elevated after 6 h of IR; treatment with reparixin significantly inhibited their production (Figures 7A,B,E). The neutrophil attractants CXCL1 and CXCL6 were also significantly increased in the serum of mice subjected to IR, but reparixin failed to reduce the levels of these chemokines (Figures 7C,D).

### Reparixin Interferes with Neutrophil Activation and Polarization

**Figure 8:**
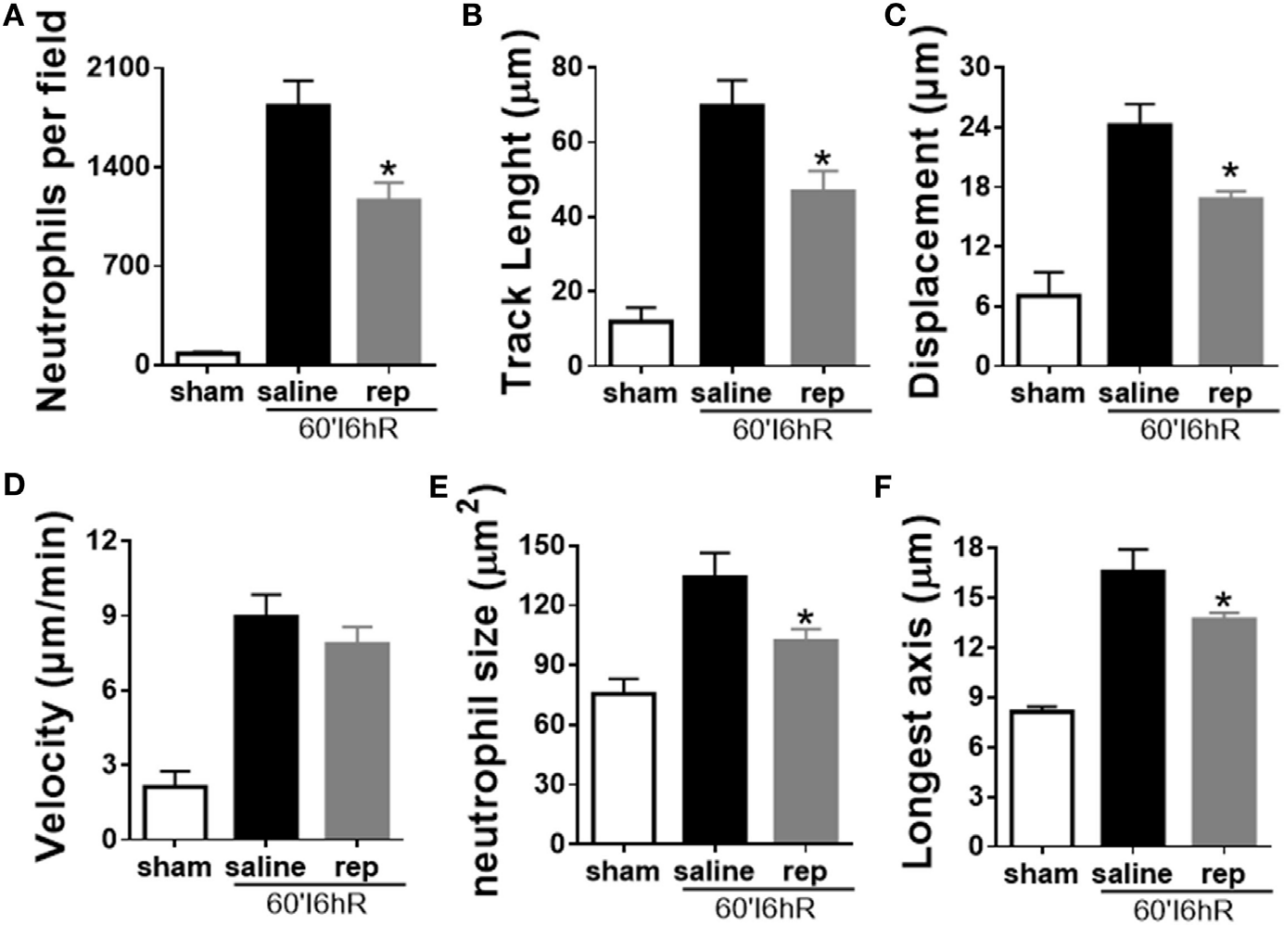
Effects of the treatment with reparixin on neutrophil tracking in mice subjected to ischemia–reperfusion (IR). Reparixin reduced neutrophil infiltration into the liver **(A)** and changed the behavior of these cells. The path length (track length) **(B)** and final displacement of neutrophils were decreased compared with animals subjected to IR and treated with saline **(C)**. There was no difference in the velocity **(D)** but reparixin reduced the size **(E)** and the longest diameter **(F)** of the neutrophils. *p < 0.05 vs relative to saline-treated livers.

We used IVM to perform a detailed investigation of parameters of leukocyte recruitment under inflammatory conditions. As reparixin treatment was able to reduce neutrophil migration and consequently liver injury, we wondered whether the lower liver injury would be associated with altered behavior of neutrophils during the course of disease. *In vivo* imaging revealed that reparixin was able to decrease neutrophil infiltration 6 h after reperfusion (Figure 8A). Moreover, neutrophils in reparixin-treated mice moved over shorter distances (Figure 8B) and showed reduced displacement (Figure 8C). However, there was no difference in the velocity of these cells (Figure 8D). Interestingly, neutrophils had smaller size (Figure 8E) and significantly less elongated shape (Figure 8F) when mice were treated with reparixin. Overall, reparixin reduced the ability of neutrophils to accumulate and migrate at sites of injury and appeared to decrease neutrophil activation.

## Discussion

Liver transplantation is a common intervention for patients with advanced liver disease. This liver surgery requires clamping of the vascular portal triad and, hence, induces ischemia, followed by reperfusion when the clamp is removed. Reperfusion injury may cause death of hepatocytes and lead to clinical complications. Hepatic IR injury is a complex phenomenon which involves numerous metabolic pathways. Although the pathophysiology of liver IR has been extensively studied for more than 40 years, its mechanisms have not yet been completely clarified (36, 37). Even if transplant survival has increased in recent years due to improved surgical techniques, as well as the development of new immunosuppressive drugs, primary graft dysfunction still represents an important cause of morbidity and mortality for transplanted patients (38). Indeed, any transplanted liver has some extent of dysfunction, since the IR lesion is an inevitable phenomenon for transplanted organs (7, 39).

When taking into account the essential functions of the liver, liver disorders have great medical importance, and without proper treatment they are often fatal (40, 41). However, there are few *in vivo* studies that describe the mechanisms of injury during liver diseases and how this modulates immune responses. Here, using confocal IVM, we provide new observations on how liver injury associated with ischemia and refusion occurs. This approach has potential for a wide range of applications to investigate the mechanism of liver IR. In this case, the opportunity to explore the movement and interaction of different cell types in their native environment is one of the major advantages of IVM.

Our studies were initiated through the standardization of the IR model, in order to establish the best time for further analysis. The animals were subjected to 60 min of ischemia, followed by different times of reperfusion. In our hands, the IR injury is severe and seems to reflect the liver status of patients with liver surgery, such as transplants. This is corroborated by tissue necrosis and ALT levels 20 times greater than baseline, which validate our model. Our data showed that neutrophils progressively infiltrated the injured liver, which was directly correlated with damage severity and liver dysfunction. Strategies to prevent recruitment of neutrophils have been shown to be beneficial in different models of IR (42, 43). Consistent with this, several studies of sterile inflammation have shown that depletion of neutrophils protect mice against liver injury (44, 45). It is suggested that strategies that limit neutrophil accumulation and/or activation may be a useful adjuvant in the treatment of ischemic disorders.

Several molecules capable of causing neutrophil migration are already known, such as CXCL1 and CXCL2 chemokines (murine homologs of human CXCL8), complement factor C5a, and leukotriene B4 (46, 47). Similarly, our data showed elevated production of CXCL1, CXCL2, and CXCL6 between 6 and 12 h after reperfusion. Previous studies demonstrated that CXC chemokines mediate neutrophil infiltration during the acute inflammatory phase of hepatic IR injury (13, 20, 48). Consistent with these observations, CXCL1 and CXCL2 have been shown to be expressed at the luminal surface of liver sinusoids around sites of necrosis. They form a chemokine gradient that guides neutrophil migration in the direction of the injury (25). In mice, hepatic expression of CXCL1 and CXCL2 increases in temporally distinct patterns after hepatic IR. The expression of CXCL2 increases in the early phase of reperfusion, before any detectable increase in neutrophil accumulation, suggesting that CXCL2 may be involved in the initial recruitment of neutrophils to the ischemic lobe (13). Neutralization of CXCL6 in an IR injury model resulted in reduced neutrophil sequestration in the liver and serum ALT levels (14). Indeed, when the chemokine is administered i.p. an increase in both circulating and peritoneal neutrophils is observed, consistent with the concept that these chemokines have a dual function, acting locally to stimulate recruitment and systemically to promote mobilization (49). When these chemokines are produced locally, they may be retained on the endothelial wall, creating a chemotactic gradient for neutrophils (25).

Chemokines act by activating G protein-coupled receptors on the surface of neutrophils. The receptor CXCR2 binds to and signals in response to murine CXCL1, CXCL2, and CXCL6. In addition, CXCL6 also interacts with murine CXCR1 (50). By recruiting and activating neutrophils, human CXCL1, CXCL2, and CXCL6 have been implicated in a wide range of diseases, including liver IR injury (15, 51). Considering the role of CXCR1/2 in neutrophil recruitment, their blockade could protect the liver of mice subjected to IR. For this reason, we have used the CXCR1/2 antagonist reparixin (DF1681B) in this model. Reparixin is a compound that inhibits the effects of CXCL1 and CXCL2 by allosteric modulation of the CXCR1/2 receptors (26). Our data demonstrated that the administration of reparixin was able to reduce neutrophil infiltration into the liver and lungs, which was associated with better liver function and histological outcome. Our results are in agreement with preliminary findings in other IR models (42, 52, 53). Reparixin inhibited the expression of TNF-α, IL-6, and CCL3, whereas it was inefficient in reducing the levels of the chemokines CXCL1 and CXCL6. This corroborates previous findings in a different model of sterile liver injury, reporting no differences in chemokine levels in mice treated with a CXCR1/2 antagonist (54). These data can be explained by the fact that although infiltrating neutrophils secrete chemokines, the primary source of chemokines in the liver may be hepatocytes, Kupffer cells, stellate cells, and sinusoidal endothelial cells. Together, these cells secrete an array of chemokines, such as CXCL1, CXCL8, CXCL9, and CXCL10 that drive leukocyte infiltration, development of inflammation, and liver injury (55–58). As reparixin acts by blocking the CXCR1/2 receptors expressed on the neutrophil surface, it does not alter chemokine production by resident cells. On the other hand, CCL3/MIP-1α is a chemokine produced only by leukocytes, especially macrophages. It can activate granulocytes, leading to acute neutrophilic inflammation (59). As the lower inflammation in reparixin-treated mice was associated with reduced infiltration of leukocytes, this may be an explanation of why the CCL3 level was decreased.

Recent advances in IVM have enabled visualization and quantification of real-time biological processes *in situ*. To better define the status of neutrophils in liver IR injury, we performed confocal intravital microscopy. Few studies have demonstrated real-time intravital neutrophil dynamics, leaving questions still open as how neutrophils behave during the sterile injury development. Besides the intense neutrophil recruitment to the liver, our *in vivo* data showed that IR caused cell death, as observed by the presence of extracellular DNA (Figure 3; see Videos S2–S4 in Supplementary Material). DNA is a DAMP, which triggers an intense immune response that can amplify the damage, when released into the extracellular environment (60). This result is in agreement with previous reports showing that during liver injury induced by acetaminophen, DNA is released from damaged hepatocytes, contributing to significantly increased systemic inflammation, liver neutrophil recruitment, and hepatotoxicity (27). Our *in vivo* investigation also showed that IR-induced polarization and activation of neutrophils during the development of liver injury. We observed that neutrophils were activated, as shown by their movement over longer distances, higher speed, formation of clusters, larger size, and increased polarity. An interesting finding of our study is that the time-course of the production of CXCL1, CXCL2, and CXCL6 in serum showed a similar trend to the time-course of neutrophil movement (track length), velocity, size, formation of clusters, and elongation of neutrophil shape. By using time-lapse TPLSM technique, Honda and colleagues have shown that CXC chemokines modulate the behavior of neutrophils during sterile liver inflammation through change in neutrophil crawling velocity (10). Indeed, McDonald and colleagues demonstrated an intravascular gradient of chemokines that contribute to drive neutrophils to the focus of liver injury. However, although this chemokine gradient is present at 100–200 µm from the site of injury, neutrophils continue to migrate into the area of necrosis (25). This indicates that from a certain point, neutrophils migrate to the necrotic area by other factors, which are independent of chemokines. This chemotactic stimulus was identified as mitochondrial *N*-formyl peptides, which can attract and activate neutrophils through the receptor FPR1 expressed on the neutrophil surface (61, 62). Studies in other animal models of liver injury have shown that antagonism of FPR1 also causes partial reduction of neutrophil recruitment. However, the strategy to block both FPR1 and CXCR1/2 receptors reduced neutrophil migration and liver damage significantly more than each treatment alone (54).

Neutrophil migration to the site of injury is a multistep process guided by a range of molecular events. However, the search for a molecular mechanism for neutrophil recruitment in the liver is complicated because a particular feature of this organ is that leukocytes do not perform rolling during migration in sinusoidal capillaries, a necessary step for the recruitment of leukocytes in most tissues (63). Instead of tampering with adhesive mechanisms, our *in vivo* data demonstrated that pharmacological antagonism of CXCR1/2 interfered with neutrophil polarization and activation in real-time, which collaborated to reduce liver injury and improve overall outcome. This reinforces the direct contribution of neutrophils to the worsening of liver IR injury. Moreover, this new approach suggests that the transition of neutrophils from a resting state to a primed state is an essential requirement for their function as competent immune cells. Several biochemical agents induce the rapid transition of neutrophils from a resting state to a polarized/activated state, leading to their transendothelial migration out of blood and recruitment to sites of infection and sterile injury in surrounding tissues (64, 65). The most impressive effect is the shape change that is observed due to the rearrangement of the cytoskeleton, which is a classical measure of cell activation. Polymerization and breakdown of actin leads to the formation and retraction of lamellipodia, which function like arms and legs of the migrating cells. Indeed, actin reorganization is required for leukocyte migration and phagocytosis. Stimulation also induces the upregulation and activation of integrins, which enable the leukocytes to adhere to the endothelial cells of the vessel wall before migrating into tissues (66, 67). Because priming and activation of neutrophils can be induced by chemical stimuli, such as chemokines, reparixin could inhibit priming or deactivate hepatic neutrophils.

Once outside the vessel, individual neutrophils often show extremely coordinated chemotaxis and cluster formation reminiscent of the swarming behavior of insects. This behavior was shown recently by Lammermann and coworkers in a model of focal ear skin damage and infected lymph nodes (68–70). They observed that once neutrophils have crossed the endothelium, arriving at the interstitial space, these cells adopt an amoeboid migration strategy, which relies mainly on contraction and protrusion of the cytoplasm, independently of adhesion molecules. This phenomenon happens in the 3D environment of the interstitium, usually composed of a meshwork of fibrillar extracellular matrix, such as collagen fibers. In this scenario, leukocyte migration is completely dependent on the actin–myosin cytoskeleton, in which leukocyte migration would happen by contraction of the cell posteriorly coupled to frontal protusion of lamellipodia, propelling the cell through the fibrillar ECM at high speed and without integrin requirement (70, 71). Interestingly, these concepts have not been applied to the liver so far, a tissue in which the reduced extravascular space is tightly packed with hepatocytes and non-parenchymal cells.

In summary, we demonstrated that IR triggers an inflammatory process in the liver with recruitment of neutrophils into the parenchyma. Neutrophil migration is greatly inhibited by treatment with a CXCR1/2 antagonist with consequent inhibition of liver damage and systemic inflammation. Importantly, we show *in vivo* that CXCR1/2 antagonists reduced not only the arrival of neutrophils in the liver but also the distance and velocity in which they migrated in this organ. Neutrophils appeared to be less activated and this was correlated with decreased local and systemic production of cytokines.

## Ethics Statement

All experiments were approved by the animal ethics committee of UFMG (CETEA/UFMG 422/15) and the ethical committee for animal experiments from KU Leuven (P111/2016).

## Author Contributions

TO, PM, PP, FA, LB, MA, and MT designed the study and TO, PP, and MT wrote the paper. All authors analyzed and interpreted the data. TO, PM, FP, and PR performed the experiments. TO and PM performed the Confocal Intravital Microscopy. FP was involved in the immunofluorescence technique. TO and PR were involved in the ELISA and qPCR techniques. All authors revised the work and approved the version to be published.

## Conflict of Interest Statement

The authors declare that the research was conducted in the absence of any commercial or financial relationships that could be construed as a potential conflict of interest.

## Supplementary Material

Videos S1–S4 Neutrophil tracking during liver injury development observed by confocal intravital microscopy. Mice subjected to sham control operation (Video 1) or ischemia–reperfusion (IR) 6 h (Video 2), IR 12 h (Video 3), and IR 24 h (Video 4) were injected intravenously with sytox orange to observe the presence of DNA as an indicator of cell death. Low-magnification intravital microscopy showed that IR induced a huge infiltration of neutrophils in the liver between 6 and 24 h of reperfusion (Lysm-eGFP-expressing cells). In addition, IR caused cell death, observed by hepatic DNA accumulation (orange), in comparison with sham-operated mice. Scale bars: 100 µm.
